# Airway Delivery of Hydrogel-Encapsulated Niclosamide for the Treatment of Inflammatory Airway Disease

**DOI:** 10.3390/ijms23031085

**Published:** 2022-01-19

**Authors:** Jiraporn Ousingsawat, Raquel Centeio, Inês Cabrita, Khaoula Talbi, Oliver Zimmer, Moritz Graf, Achim Göpferich, Rainer Schreiber, Karl Kunzelmann

**Affiliations:** 1 Physiological Institute, University of Regensburg, University Street 31, 93040 Regensburg, Germany; Jiraporn.Ousingsawat@vkl.uni-regensburg.de (J.O.); Raquel.Martins-Centeio@vkl.uni-regensburg.de (R.C.); inesmscabrita@gmail.com (I.C.); Khaoula.Talbi@vkl.uni-regensburg.de (K.T.); Rainer.Schreiber@vkl.uni-regensburg.de (R.S.); 2 Department of Pharmaceutical Technology, University of Regensburg, 93040 Regensburg, Germany; Oliver.Zimmer@chemie.uni-regensburg.de (O.Z.); Moritz.Graf@chemie.uni-regensburg.de (M.G.); Achim.Goepferich@chemie.uni-regensburg.de (A.G.)

**Keywords:** TMEM16A, TMEM16F, asthma, inflammatory airway disease, COVID-19, hydrospheres, nanospheres, niclosamide

## Abstract

Repurposing of the anthelminthic drug niclosamide was proposed as an effective treatment for inflammatory airway diseases such as asthma, cystic fibrosis, and chronic obstructive pulmonary disease. Niclosamide may also be effective for the treatment of viral respiratory infections, such as SARS-CoV-2, respiratory syncytial virus, and influenza. While systemic application of niclosamide may lead to unwanted side effects, local administration via aerosol may circumvent these problems, particularly when the drug is encapsulated into small polyethylene glycol (PEG) hydrospheres. In the present study, we examined whether PEG-encapsulated niclosamide inhibits the production of mucus and affects the pro-inflammatory mediator CLCA1 in mouse airways in vivo, while effects on mucociliary clearance were assessed in excised mouse tracheas. The potential of encapsulated niclosamide to inhibit TMEM16A whole-cell Cl^−^ currents and intracellular Ca^2+^ signalling was assessed in airway epithelial cells in vitro. We achieved encapsulation of niclosamide in PEG-microspheres and PEG-nanospheres (Niclo-spheres). When applied to asthmatic mice via intratracheal instillation, Niclo-spheres strongly attenuated overproduction of mucus, inhibited secretion of the major proinflammatory mediator CLCA1, and improved mucociliary clearance in tracheas ex vivo. These effects were comparable for niclosamide encapsulated in PEG-nanospheres and PEG-microspheres. Niclo-spheres inhibited the Ca^2+^ activated Cl^−^ channel TMEM16A and attenuated mucus production in CFBE and Calu-3 human airway epithelial cells. Both inhibitory effects were explained by a pronounced inhibition of intracellular Ca^2+^ signals. The data indicate that poorly dissolvable compounds such as niclosamide can be encapsulated in PEG-microspheres/nanospheres and deposited locally on the airway epithelium as encapsulated drugs, which may be advantageous over systemic application.

## 1. Introduction

Previous work showed that the inhibition of the Ca^2+^ activated Cl^−^ channel TMEM16A inhibits excessive mucus production in inflammatory airway diseases such as asthma and cystic fibrosis (reviewed in [1]). TMEM16A is upregulated in cystic fibrosis and asthma, which accompanies goblet cell metaplasia (in asthma) and goblet cell hyperplasia (in cystic fibrosis) and mucus hypersecretion [2,3]. TMEM16A is also upregulated by bacterial components [4,5,6]. Upregulation of TMEM16A is predominant in mucus-producing cells located in airway submucosal glands, and to a lesser degree in ciliated epithelial cells [2,6,7]. Moreover, TMEM16A and the related protein TMEM16F contribute to the ferroptotic death of airway epithelial cells, caused by *Pseudomonas aeruginosa (P. aeruginosa)*-induced plasma membrane lipid peroxidation [8,9]. TMEM16F was also shown to be central to syncytia formation observed in the lungs of patients with severe COVID-19 [10]. The syncytia formation is triggered when SARS-CoV-2 spike (S) proteins bind to angiotensin converting enzyme type 2 (ACE2) receptors on the surface of neighboring cells [11]. This may contribute to viral dissemination, immune evasion, and inflammatory response. Thus, TMEM16 proteins are highly relevant therapeutic targets in asthma, cystic fibrosis, and viral lung infections caused by SARS-CoV-2, respiratory syncytial virus, or influenza [10,12]. Although these predominantly airway-located diseases are of very different origin, involving distinct cytokines and patho-mechanisms, they have a participation of one (TMEM16A) or two (TMEM16A and TMEM16F) members of the TMEM16-family of proteins in common. Therefore, a drug that inhibits the activity of several TMEM16-members by means of lowering Ca^2+^-dependent activation may be useful in the therapy of these airway diseases [13].

The anthelminthic drug niclosamide and related substances such as nitazoxanide were proposed as repurposed drugs for the treatment of asthma, cystic fibrosis, and chronic obstructive pulmonary disease [1,14,15]. Niclosamide is a potent inhibitor of TMEM16A and TMEM16F [13,14,15] and causes bronchorelaxation in vivo and ex vivo. It potently inhibited mucus production and mucus secretion, and strongly suppressed the release of the cystic fibrosis-typical interleukin IL-8 [6,14,15]. Moreover, it was also shown to attenuate lung vascular remodeling in experimental pulmonary arterial hypertension [16]. During the screening of libraries containing FDA-approved drugs, niclosamide was also identified as a potent inhibitor of the replication of severe acute respiratory corona virus [17]. Niclosamide inhibits endosomal acidification, and may also act via additional mechanisms, thereby inhibiting virus uptake and virus replication [18,19,20]. It acts as a proton shuttle, which explains its broad antiviral activity [21]. 

TMEM16A and other TMEM16 family members also control intracellular Ca^2+^ levels [22,23]. As SARS-CoV-2 and other viruses hijack intracellular Ca^2+^ signaling to benefit their infection and replication, the inhibition of intracellular Ca^2+^ signaling by the TMEM16-inhibitor niclosamide may contribute to its antiviral activity [24,25,26]. Currently, niclosamide and the related compound nitazoxanide are under investigation in numerous clinical trials to determine their efficacy in the treatment of COVID-19 (clinicaltrials.org) [27,28,29,30].

Niclosamide has very low water solubility between 0.23 and 1.6 μM/mL under ambient conditions. This is the reason for poor intestinal absorption causing only low plasma levels when applied orally [31]. To enhance its bioavailability, nanoparticle formulations were developed and injected into rats intramuscularly, which reached peak plasma concentrations of about 4.3–9.4 µM, when applied at 50–300 mg/kg [32]. The systemic application of encapsulated niclosamide might cause high systemic plasma levels, which could result in unwanted side effects. We therefore examined the effects of a local application of encapsulated niclosamide to mouse airways. In a similar approach, the successful delivery of antibiotics to airways using nanocarriers has been reported previously [33]. We found that similar to intratracheal application of the dissolved compound [15], niclosamide encapsulated in polyethylene glycol hydrogels shows potent mucus-suppressing and anti-inflammatory effects, and improves mucociliary clearance. The effects induced by intratracheal application in vivo could be reproduced in vitro. Along with these additional results, which indicate the inhibition of TMEM16A by suppression of intracellular Ca^2+^ signals, we propose niclosamide encapsulated in PEG-microspheres/nanospheres as an effective topical treatment of inflammatory airway disease.

## 2. Results

### 2.1. Niclosamide-Loaded Microspheres and Nanospheres (Niclo-Spheres) Inhibit Mucus Production in Airways of Asthmatic Mice 

**Figure 1:**
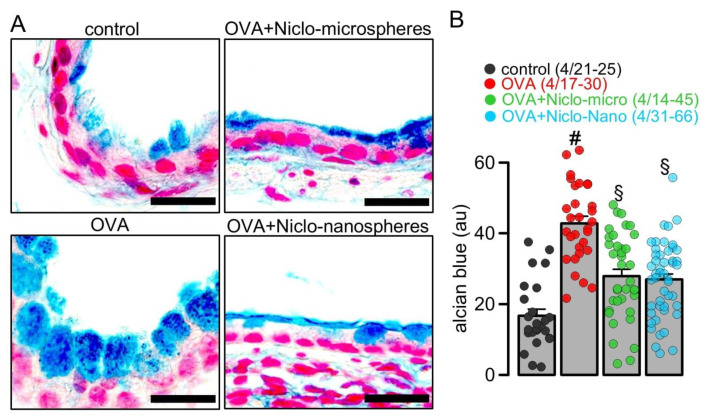
*Inhibition of mucus production by Niclo-spheres of micrometer and nanometer size.* (**A**) Airway mucus staining by alcian blue in control mice and asthmatic mice sensitized with ovalbumin (OVA). Intratracheal instillation of niclosamide (Niclo)-loaded microspheres (0.98 mg; 30 µM niclosamide) or nanospheres (0.196 mg; 30 µM niclosamide) strongly reduced mucus production in bronchi of asthmatic mice. Bar = 50 µm. (**B**) Summary of experiments as shown in (**A**). Mean ± SEM (number of animals/number of bronchi analyzed). # significant difference from control. § significant difference from OVA (*p* < 0.05, ANOVA).

Hydrogel microparticles with a molecular mass of 10,000 Da were generated with an approximate spherical particle size of the rehydrated particle between 4 and 45 µm. PEG-coated nanospheres were prepared by sequential nanoprecipitation [34], leading to particles with hydrodynamic diameters of around 97–125 nm. Ovalbumin (OVA)-sensitization was used as a common method to induce allergic asthma in rodents [35]. We detected goblet cell metaplasia and strong upregulation of mucus production in the airways of OVA-sensitized mice (Figure 1A). Intratracheal application of Niclo-spheres (containing 30 µg niclosamide) strongly reduced mucus production in the airways of OVA-sensitized mice (Figure 1A,B). The effects of niclosamide encapsulated in microspheres or nanospheres were comparable. Thus, niclosamide is efficient in suppressing mucus production when applied as a dissolved powder, as demonstrated earlier [6,15], or when deposited as Niclo-spheres. Although no direct comparison was conducted regarding the efficacy of Niclo-spheres versus dissolved niclosamide, the effect reported earlier for dissolved niclosamide [15] and the present data appear quite comparable.

### 2.2. Accumulation of mCLCA1 in Club Cells by Treatment with Niclo-Spheres 

**Figure 2:**
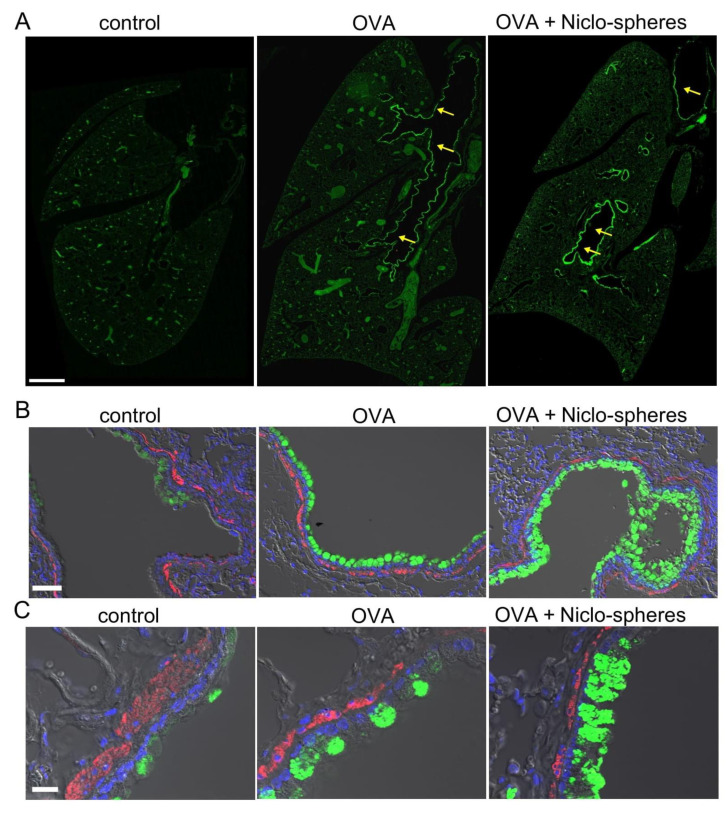
*Accumulation of mCLCA1 in club cells by treatment with Niclo-spheres*. (**A**) Whole mouse lung immunofluorescence obtained by stitching microscopy. mCLCA1 is expressed particularly in larger bronchi (yellow arrows), but not in small bronchioles and the peripheral lung tissue of asthmatic (ovalbumin (OVA)-treated) mice, and OVA-mice treated with Niclo-spheres. Intratracheal instillation of niclosamide (Niclo)-loaded spheres (Niclo-spheres; 0.196 mg; 30 µM/5 days) induced additional accumulation of mCLCA1 in club cells. Bar = 1 mm. (**B**,**C**) mCLCA1 (green) and smooth muscle α-actin (red) at lower (**B**; bar = 50 µm) and higher (**C**; bar = 20 µm) magnification. Nuclei staining by DAPI (blue). Low expression of mCLCA1 under control conditions is upregulated in OVA-treated mice. Further accumulation of CLCA1 is observed in club cells from mice treated with Niclo-spheres; 2–3 animals for each series.

CLCA1 (Calcium-activated chloride channel regulator 1) is upregulated in airway epithelial cells during airway inflammation [36]. CLCA1 is a secreted metalloproteinase that modulates the function of TMEM16A [37], drives mucus production [38], and shows additional pleiotropic effects during airway inflammation [39]. We analyzed CLCA1 in the airways of OVA-sensitized and healthy control animals and found enhanced expression of CLCA1 in the airways of OVA-treated animals. An in-depth analysis of CLCA1 expression was performed using stitching microscopy [40]. The release of CLCA1 from secretory cells appeared to be inhibited by Niclo-spheres (30 µM for 5 days intratracheal application) and thus accumulated in secretory (club) cells (Figure 2A–C). Thus, niclosamide inhibits the release of CLCA1, similar to its inhibitory effect on mucus secretion described earlier [5]. The data suggest encapsulated niclosamide as an effective anti-inflammatory treatment in asthma and other inflammatory airway diseases.

### 2.3. Niclo-Spheres Improve Mucociliary Clearance in Asthmatic Mice

**Figure 3:**
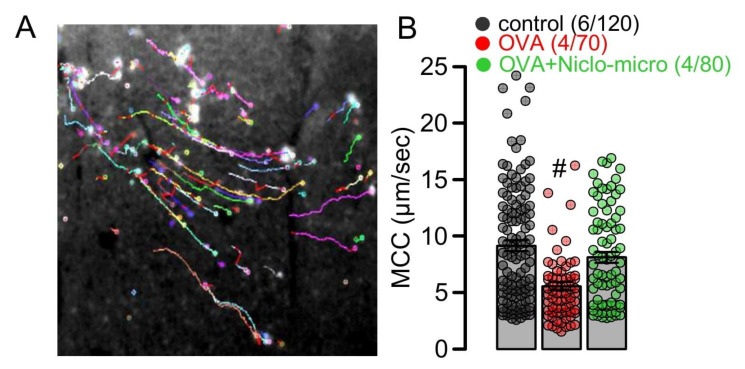
*Effect of Niclo-spheres on mucociliary clearance.* (**A**) Image from particle tracking in mouse trachea ex vivo, as a measure mucociliary clearance. (**B**) Summary for particle movement (µm/s) as a measure for mucociliary clearance (MCC) in tracheas ex vivo indicates that niclosamide-microspheres (0.98 mg; 30 µM) augment MCC that was reduced in tracheas of OVA-treated animals. Mean ± SEM (number of animals/number of measurements). # significant difference from control (*p* < 0.05, ANOVA).

Niclosamide is a potent inhibitor of the Ca^2+^ activated Cl^−^ channel TMEM16A [13,14,15]. TMEM16A has been claimed to contribute to Cl^−^ secretion by the airway epithelium and is assumed to enhance the airway surface liquid layer and to increase mucociliary clearance [41]. Thus, the inhibition of TMEM16A may counteract the inhibition of airway inflammation and mucus production. We used particle tracking in mouse tracheas ex vivo to measure the effect of niclosamide on mucociliary clearance (Figure 3A). The data show reduced mucociliary clearance in OVA-sensitized animals, when compared to sham-treated animals (control). Treatment with microsphere-encapsulated niclosamide enhanced particle transport, almost to values found in control animals (Figure 3B). This result suggests that niclosamide, while possibly inhibiting the activity of TMEM16A in fluid-secreting cells [6], has no negative effects on mucus transport, but rather improves mucociliary clearance.

### 2.4. Activation of the Ca^2+^ Activated Cl^−^ Channel TMEM16A Is Inhibited by Niclo-Spheres, but Not by Empty PEG Spheres 

**Figure 4:**
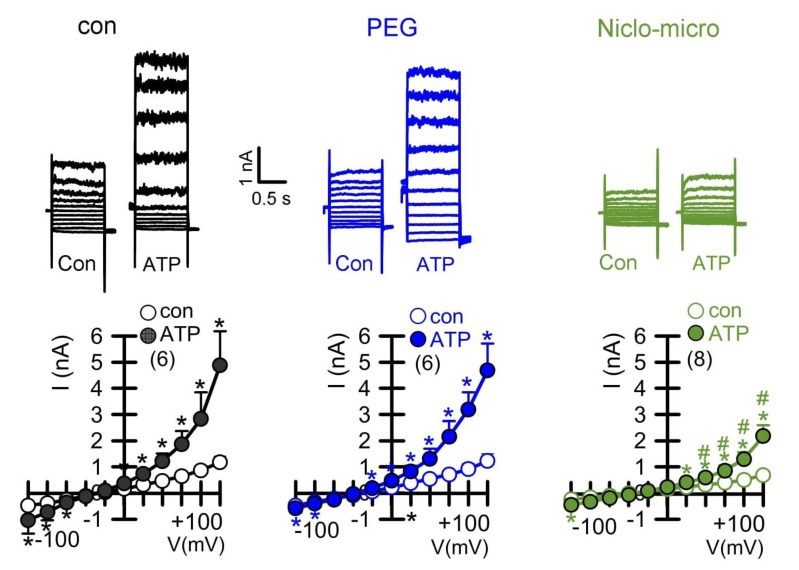
*Activation of the Ca^2+^ activated Cl^−^ channel TMEM16A in CFBE human bronchial epithelial cells and inhibition by Niclo-spheres, but not by polyethylene glycol.* Whole-cell current overlays (upper panels) and corresponding current/voltage relationships (lower panels). Activation of whole cell currents by purinergic stimulation (ATP; 100 µM) in CFBE airway epithelial cells was comparable in non-treated control cells (con) and in cells exposed to empty polyethylene glycol microspheres (PEG), but was potently inhibited by microsphere-encapsulated niclosamide (Niclo-micro, 1 µM). The inhibitor of Ca^2+^-activated KCNN4 K^+^ channels, TRAM-34 (100 nM), was present in all patch clamp experiments to avoid unwanted activation of Ca^2+^ activated K^+^ channels. Mean ± SEM (number of cells). * significant activation by ATP (*p* < 0.05; paired *t*-test). # significant difference when compared to con or PEG (*p* < 0.05; unpaired *t*-test).

**Figure 5:**
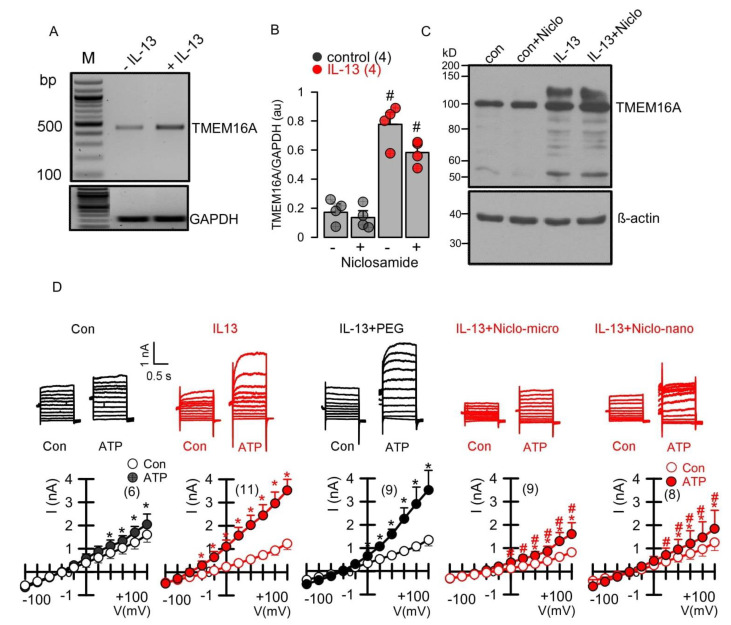
*Enhanced expression of TMEM16A in Calu-3 human airway submucosal epithelial cells by IL-13 and inhibition of TMEM16A currents by Niclo-spheres.* (**A**,**B**) RT-PCR analysis demonstrating enhanced expression of TMEM16A after incubation by IL-13 (20 ng/mL; 72 h) in the absence or presence of niclosamide (1 µM) (number of reactions). (**C**) Western blot indicating upregulation of TMEM16A-expression by IL-13 in the absence or presence of niclosamide. Note that expression of the glycosylated form of TMEM16A (upper band) is enhanced upon exposure to IL-13. Western blots were performed in triplicates. (**D**) Whole-cell current overlays (upper panels) and corresponding current/voltage relationships (lower panels). Activation of whole cell currents by purinergic stimulation (ATP; 100 µM) was enhanced by IL-13. Niclosamide encapsulated in microspheres (1 µM) or nanospheres (1 µM) strongly inhibited activation of TMEM16A currents by ATP. Empty spheres (PEG only) did not inhibit TMEM16A currents. The inhibitor of Ca^2+^-activated KCNN4 K^+^ channels, TRAM-34 (100 nM), was present in all patch clamp experiments, to avoid potential activation of Ca^2+^ activated K^+^ channels. Mean ± SEM (number of cells). * significant activation by ATP (*p* < 0.05; paired *t*-test). # significant difference when compared to the absence of microspheres or nanospheres (*p* < 0.05; unpaired *t*-test).

Previous studies have shown that niclosamide dissolved in an aqueous solution inhibits TMEM16A ion currents [13,14,15]. We examined whether encapsulated niclosamide also inhibits TMEM16A currents, when applied acutely to CFBE human bronchial epithelial cells. To this end, cells were stimulated with ATP to activate TMEM16A. The application of empty polyethylene glycol microspheres (PEG) did not inhibit ATP-activated TMEM16A currents. In contrast, the application of niclosamide-containing microspheres potently suppressed ATP-activated TMEM16A currents (Figure 4). We further examined whether Ca^2+^ activated TMEM16A currents present in Calu-3 human airway submucosal cells are also inhibited by encapsulated niclosamide. To that end, the expression of TMEM16A was enhanced by the exposure of the cells to interleukin IL-13. IL-13 augmented the expression of TMEM16A mRNA (Figure 5A,B) and the TMEM16A protein (Figure 5C). While a small whole-cell current was activated by ATP in control Calu-3 cells, the exposure to IL-13 strongly augmented the activation of TMEM16A currents. The activation of TMEM16A was not affected by empty microspheres (PEG), but was significantly inhibited by niclosamide encapsulated in microspheres or nanospheres (Figure 5D). 

### 2.5. Niclo-Spheres Inhibited Expression of MUC5AC in Calu-3 Human Airway Submucosal Epithelial Cells 

**Figure 6:**
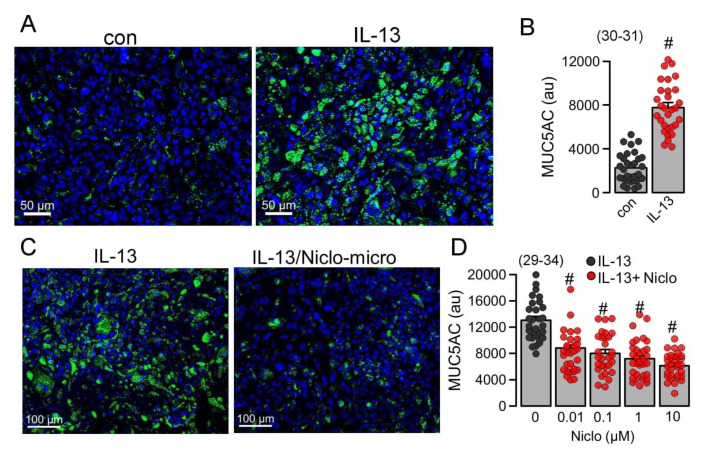
*Inhibition of MUC5AC in Calu-3 human airway submucosal cells by Niclo-spheres.* (**A**) Induction of MUC5AC expression (green fluorescence) in Calu-3 cells. Bar = 50 µm. (**B**) Quantitative analysis of MUC5AC expression indicates significant upregulation by IL-13 (20 ng/mL/72 h). (**C**) Inhibition of MUC5AC expression by niclosamide-loaded microspheres (Niclo-micro). Bar = 100 µm. (**D**) Concentration-dependent inhibition of MUC5AC expression by niclosamide-loaded microspheres. Mean ± SEM (number of sections analyzed). # significant difference when compared to control (*p* < 0.05; unpaired *t*-test) or IL-13 (*p* < 0.05; ANOVA).

We further examined if the expression of the mucin MUC5AC, induced by the Th2-inflammatory mediator IL-13, is inhibited by Niclo-spheres. MUC5AC expression in Calu-3 cells was strongly enhanced by IL-13. (Figure 6A,B). The application of Niclo-spheres strongly attenuated the expression of MUC5AC (Figure 6C). Notably, even concentrations of Niclo-spheres as low as 10 nM potently inhibited the expression of MUC5AC (Figure 6D). This is in accordance with earlier studies showing the potent inhibition of TMEM16A by dissolved niclosamide and related compounds at low nanomolar concentrations [14,15]. The data confirm the comparable effects of dissolved niclosamide [15] and encapsulated niclosamide (present data).

### 2.6. Niclosamide Inhibits Mucus Production, CLCA1-Secretion and TMEM16A Probably by Blocking Intracellular Ca^2+^ Signals 

**Figure 7:**
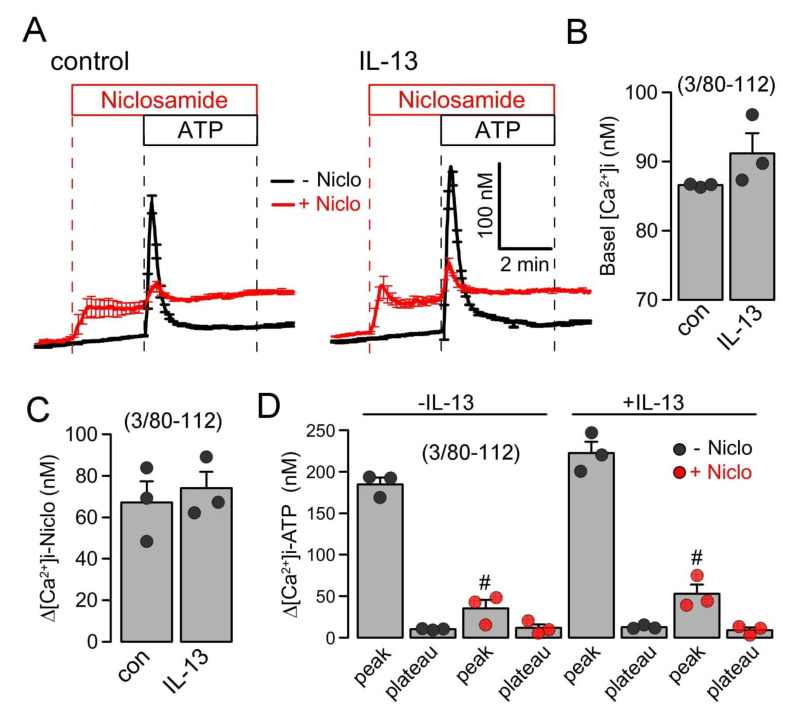
*Inhibition of intracellular Ca^2+^ signals in Calu-3 cells by Niclo-spheres.* (**A**) Summary traces for the intracellular Ca^2+^ concentration measured in control Calu-3 cells (left) and cells treated with IL-13 (20 ng/mL/72 h; right). (**B**) Summary of basal Ca^2+^ in treated and untreated cells. (**C**) Summary for the increase in intracellular Ca^2+^ by niclosamide (5 µM). (**D**) Summary of ATP (100 µM)-induced increase in cytosolic Ca^2+^. IP_3_-induced store release Ca^2+^ (peak) but not store operated Ca^2+^ influx (SOCE; plateau) was strongly attenuated by niclosamide. Mean ± SEM (number of independent series/number of cells analyzed). # significant difference when compared to control (*p* < 0.05; unpaired *t*-test) or IL-13 (*p* < 0.05; ANOVA).

Intracellular Ca^2+^ concentrations were measured in control Calu-3 cells and cells treated with IL-13, using the Ca^2+^ dye Fura2-AM. IL-13 slightly, but not significantly, augmented basal Ca^2+^ and ATP-induced Ca^2+^ increase. The acute application of niclosamide augmented basal Ca^2+^ levels and almost abolished the ATP-induced rise in intracellular Ca^2+^ (Figure 7). Niclosamide probably depletes endoplasmic reticulum Ca^2+^ stores, likely by the inhibition of the sarcoplasmic-endoplasmic reticulum Ca^2+^-ATPase (SERCA) and by the inhibition of store-operated Ca^2+^ entry (SOCE) [22]. Taken together, the present data suggest that niclosamide, encapsulated in microspheres or nanospheres, inhibits airway mucus production, attenuates airway inflammation, and inhibits the activation of TMEM16A by the suppression of intracellular Ca^2+^ signals.

## 3. Discussion

Polyethylenimine-based non-viral gene delivery systems have been developed to target drugs efficiently [42]. Thus, niclosamide-encapsulated nanocarriers were shown to inhibit the growth of cancer cells [43]. PEG-formulations of poorly absorbable niclosamide were shown to provide better bioavailability, which is highly relevant for its application as an anti-cancer drug and probably useful in the treatment of COVID-19 [44]. It was shown that microgels unload their cargo over a 6–14-day period without showing significant cytotoxicity. Furthermore, recognition by alveolar macrophages was considerably lower than for polystyrene control particles [45]. Thus, airway deposition of encapsulated drugs appears ideal to maintain high effective local drug concentrations, without the need of repetitive applications. To that end, further chemical modification of hydrogels, by modification with ligands of mucosal surface receptors, is likely to increase tissue targeting, thereby enhancing specificity and local drug deposition [46]. Along this line, nanospheres conjugated with hyaluronic acid, a ligand of CD44, were designed to target siRNA to the airway epithelium [47]. PEG nanospheres penetrate well airway mucus. This would be an advantage particularly for the treatment of cystic fibrosis and asthma, which show the accumulation of copious amounts of mucus in the airways [48]. In fact, the PEG-formulation of drugs was shown to improve efficacy in other studies [49,50]. Because the expression of CD44, the receptor for hyaluronic acid (HA), is enhanced in the airway epithelium of asthmatic subjects, the conjugation of Niclo-spheres to HA may further enhance the targeting of airway epithelial cells [51]. We also found an upregulation of CD44 in the inflamed airways of humans and mice in the present study (Supplementary Figure S1). However, because the upregulation of HA was observed predominantly in the basolateral (but not apical) membrane, conjugation of Niclo-spheres with an inactive form of angiotensin II (ATII) might be more effective. This could also be useful in the treatment of COVID-19. SARS-CoV-2 binds via its spike protein to the angiotensin-converting enzyme 2 (ACE2), an enzyme receptor attached to the apical membrane of airway epithelial cells [52]. Conjugation with inactive ATII may improve the targeting of niclosamide to the site of infection and, in addition, may compete with the binding of the virus.

Niclosamide was suggested to induce a number of positive effects in asthma and cystic fibrosis lung disease by inhibiting TMEM16 proteins [6,14,15]. It was also shown to inhibit the replication of severe acute respiratory corona virus [17], and to block SARS-CoV-2 spike-protein-induced syncytia formation [10]. COVID-19 is characterized by abnormal activation of the inflammatory response and rapid deterioration of lung function due to alveolar edema [53]. Infected pneumocytes in the lungs of COVID-19 patients show abnormal morphology and frequent multinucleation. The generation of these cellular syncytia is caused by the spike protein, which, during the process of cell fusion, activates the phospholipid scramblase TMEM16F [10,54]. Activated TMEM16F disturbs the plasma membrane asymmetry, i.e., the polarized distribution of plasma membrane phospholipids, which leads to the activation of platelets, membrane shedding, and the activation of macrophages [55,56,57]. TMEM16A is likely to contribute to these processes because it supports the activation of TMEM16F, probably by ER-tethering to the plasma membrane and facilitating an intracellular Ca^2+^ increase [58]. Thus, TMEM16 proteins have a significant role in viral lung diseases, which should be explored in more detail in future studies. Although the present results demonstrate a pronounced reduction of mucus production and suggest the attenuated release of CLCA1 into the airway lumen, the available data are limited to mouse airways. Moreover, the encapsulated drug has been applied by intratracheal instillation, and so far, no data are available for aerosol application, which probably would be used for topical drug application in human airways. Finally, studies on the long-term application will be required to fully assess potential unwanted effects. Taken together, the results from the present study suggest the use of Niclo-spheres in the treatment of COVID-19 and other viral respiratory infections as well as chronic inflammatory diseases such as cystic fibrosis and asthma.

## 4. Material and Methods

### 4.1. Preparation of Niclosamide-Loaded PEG-Spheres 

For the fabrication of hydrogel microparticles, eight-armed poly(ethylene glycol) macromonomers with a molecular mass of 10,000 Da (8armPEG_10k_) were functionalized at its hydroxyl groups with norbornene moieties via an ester bond (8armPEG_10k_-e-NB), as described earlier [59]. A hydrolytically cleavable PEG-linker with thiol-groups (PEG_1k_-e-MPA) was prepared by end-functionalizing PEG_1k_ in a similar manner to an esterification method described previously [59]. In brief, PEG, mercaptopropionic acid (8-fold molar excess of PEG OH-groups) and 0.4 mmol p-toluenesulfonic acid were dissolved in toluene. The reaction mixture was subsequently refluxed in a Dean–Stark apparatus under stirring for 24 h. Toluene was removed via a rotary evaporator, and the product was precipitated thrice in ice cold diethyl ether. 1H NMR showed a conversion rate of 93.3%, whereas the total yield amounted to 92.6%. A stock niclosamide suspension (Thermo Fisher Scientific, Waltham, USA) was prepared via wet-milling of 2.1 g NCL with 0.21 g Tween 20 and 4.5 mL MilliQ in a 25 mL stainless-steel milling cup with stainless-steel balls (Ø = 1 mm) [60]. Cycles (*n* = 5) of milling for 30 min at 600 rpm were applied with a 30 min cooling time in between. The resulting suspension was centrifuged down and the residue was resuspended in MilliQ by sonication for 30 min. The resulting Particle size distribution was determined by laser diffraction measurement (Mastersizer 2000, Malvern Panalytical Ltd, Malvern, United Kingdom) (d10 = 0.106 µm/d50 = 0.543 µm/d90 = 3.937 µm), and the NCL-content of 91.5 mg/mL was measured via UV-Vis (Kontron Uvikon 941, Goebel Instrumentelle Analytik GmbH, Au in der Hallertau, Germany) at 345 nm. A bulk hydrogel containing niclosamide particles was prepared by crosslinking a solution of 72.25 mg 8armPEG_10k_-e-Norb and 28.9 mg PEG_1k_-e-MPA in 0.739 mL MilliQ with Eosin Y (0.1 mM) and additional 110.5 µL niclosamide stock by irradiation with green light (525 nm) for 20 min [61]. Freeze drying for 125 h with a starting shelf temperature of −40 °C and 0.01 mbar and subsequently 5 h at a 10 °C shelf temperature yielded a dry gel. For the preparation of inhalable PEG-hydrogel particles, bulk freeze-dried hydrogels were cut into pieces of about 1 mm edge length and mixed with LH 70 lactose-particles (MEGGLE Group GmbH, Wasserburg am Inn, Germany) to yield 10% wt. PEG-content. The resulting mixture was pre-milled in a Retsch PM100 planetary ball mill, using a 25 mL stainless-steel milling cup with 7 stainless-steel balls (Ø = 10 mm). The powders underwent 5 cycles of 2:15 min milling at 600 rpm and 14 min cooling time. Pre-ground powders were finally milled 3 times in a McOne (Jetpharma SA, Balerna, Switzerland) lab scale jet-mill at 12–13 bar venturi pressure and 9–10 bar grinding pressure (Linde 4.0 nitrogen). Particle size distributions of rehydrated hydrogel particles were determined as d10 = 4.332 µm, d50 = 11.310 µm, and d90 = 45.932 µm via laser diffraction (Malvern Mastersizer 2000). The refractive index of PEG-particles was chosen as 1.430 and the absorption as 0.01 based on previous investigations [59].

PEG-coated nanospheres were prepared via previously described sequential nanoprecipitation [34] of niclosamide and methoxy-terminated poly(ethylene glycol)_5k_-b-poly(D,L-lactide)_10k_ (MeO-PEG_5k_-PLA_10k_). MeO-PEG_5k_-PLA_10k_ synthesis was previously published [62,63]. First, we prepared a 0.01 mg/mL stock solution of niclosamide in DMSO:DMF (1:1 *v/v* ratio) (NIC-ss) and 0.02 mg/mL stock solutions of niclosamide and MeO-PEG_5k_-PLA_10k_ in DMSO:DMF (1:1 *v/v* ratio), which were mixed in a 1:1 ratio (NIC/PEG-PLA-ss). To each of three 50 µL samples of NIC-ss (uncoated nanospheres) or NIC/PEG-PLA-ss (PEG-coated nanospheres), 950 µL milliQ H_2_O was added and the reaction was thoroughly homogenized. The three reactions were mixed and up-concentrated using a 30 kDa cut-off Microsep advance centrifugal device (2000 rcf, 15 min). This process was repeated ten times for further up-concentration of the nanospheres. The preparation yielded approximately 300 µL of the niclosamide-nanosphere solution equivalent to 150 µM niclosamide. Nanospheres were characterized using dynamic light scattering on a ZetaSizer Nano ZS using 633 nm He-Ne laser at a backscatter angle of 173° (Malvern Instruments GmbH, Lappersdorf, Germany). The hydrodynamic diameter (*d_h_*) and polydispersity-index (PDI) were measured for uncoated nanospheres (*d_h_* = 97.3 ± 3.8 nm, PDI = 0.09 ± 0.03; mean ± std) as well as for PEG-coated nanosphere before (*d_h_* = 91.1 ± 7.4 nm, PDI = 0.16 ± 0.04; mean ± std) and after up-concentration (*d_h_* = 125 ± 3.1 nm, PDI = 0.25 ± 0.02; mean ± std).

### 4.2. Animals and Treatments 

Allergen challenge of mice has been described previously [35]. In brief, mice were sensitized to ovalbumin (OVA; Sigma-Aldrich, St. Louis, MO, USA) by an intraperitoneal (I.P.) injection of 100 µg OVA in 100 µL aluminum hydroxide gel adjuvant (InvivoGen, San Diego, CA, USA) on days 0 and 14. At days 21 to 23, mice were anesthetized (ketamine 90–120 mg/kg and xylazine 6–8 mg/kg) and challenged to OVA by intratracheal (I.T.) instillation of 50 µg OVA in 100 µL saline with or without Niclosamide or Niclosamide-enclosing micro- and nanoparticles for a final concentration of 30 µM Niclosamide. Control mice were sham sensitized with aluminum hydroxide gel and challenged to saline by I.T. instillation. The allergen reaction was allowed to develop for 72 h, while all niclosamide instillations were maintained for days 24 and 25. Animals were sacrificed and tissues were collected on day 26. All animal experiments complied with the guidelines for animal research and were carried out in accordance with the ‘United Kingdom Animals Act, 1986’ and associated guidelines, as well as EU Directive 2010/63/EU for animal experiments. All animal experiments were approved by the local Ethics Committee of the Government of Unterfranken/Wurzburg (AZ: 55.2.2-2532-2-1359-15) and were conducted according to the guidelines of the American Physiologic Society and German Law for the Welfare of Animals.

### 4.3. Cell Culture and Treatments 

All cells were grown at 37 °C in a humidified atmosphere with 5% CO_2_. Calu-3 human airway epithelial cells were grown in DMEM/Ham’s F-12 with L-Glutamine medium supplemented with 10% (*v/v*) fetal bovine serum (FBS), 1% (*v/v*) L-glutamine 200 mM, and 1% (*v/v*) HEPES 1 M (all from Capricorn Scientific, Ebsdorfergrund, Germany). Cells were treated with IL-13 (20 ng/mL; Enzo Life Sciences, Lörrach, Germany) for 72 h in Opti-MEM, and the treatment was refreshed every day. The human airway cell line CFBE was cultured as described previously [64]. Cells were treated with niclosamide encapsulated in polyethylene glycol (PEG) hydrogel microspheres. The final concentration of niclosamide was calculated on the basis that niclosamide accounted for 1% of the microsphere’s weight and the equivalent niclosamide concentration in nanospheres. 

### 4.4. MUC5AC and Alcian Blue Analysis 

Mouse airways were fixed by transcardial perfusion and lung perfusion by tracheal instillation via tracheostomy of a fixative solution containing 4% PFA in PBS. Tissues were left in a fixative solution overnight and embedded in paraffin the next day. Furthermore, 5 µm cuts were deparaffinized, stained with standard Alcian blue solution, and counterstained with Nuclear Fast Red solution (Sigma-Aldrich, St. Louis, MO, USA). After dehydration and clearing steps, whole mouse lungs or sections were mounted in DePeX mounting medium (SERVA Electrophoresis, Heidelberg, Germany). Stainings were assessed by light microscopy. Mucus-stained areas were determined using ImageJ. For MUC5AC stainings, Calu-3 airway cells seeded onto glass coverslips were fixed with 4% PFA/PBS for 10 min at room temperature, washed in PBS with Ca^2+^ and Mg^2+^ incubated in 0.5% Triton X-100/PBS for 10 min at room temperature, washed, blocked with 1% BSA/PB^+^ for 40 min at room temperature, incubated with mouse monoclonal anti-MUC5AC antibody (1:300 in 1%BSA/PBS; #ab3649; Abcam, Cambridge, UK) for 1 h at 37 °C, washed, incubated with Alexa Fluor 488-labeled donkey anti-mouse IgG (1:300 in 1%BSA/PBS, Invitrogen, Carlsbad, CA, USA), and counterstained with Hoe33342 (1:200) for 1 h at room temperature. Cells were then washed and mounted in a fluorescence mounting medium. MUC5AC-staining was quantified using ImageJ FIJI version 1.53e (National Institutes of Health, Bethesda, MD, USA) [65].

### 4.5. Immunocytochemistry of mCLCA1 and CD44 

Mouse lungs were fixed by transcardial and lung perfusion fixation. Mouse airways were embedded in paraffin, and tissue sections (5 µm) were stained using the standard protocol for immunofluorescence. CD44 and CLCA1 were detected using rat anti-CD44 conjugated with PE-Cyanine7 (Invitrogen, Dreieich, Germany) and rabbit anti-mouse Clca3 antibody (ab46512, Abcam, Berlin, Germany), respectively. The nucleus was counterstained with 5 µM Hoe33342 (Thermo Fisher Scientific, Darmstadt, Germany). Immunofluorescence was detected with an Axio Observer microscope equipped with Axiocams 503 mono, ApoTome.2, and ZEN 3.0 (blue edition) software (Zeiss, Oberkochen, Germany). Stitching microscopy was performed using a motorized Axio Observer and Zen software [40].

### 4.6. Microscopic Measurements of the Mucociliary Clearance 

Mucociliary clearance ex vivo was performed by tracking fluorescence particles’ movement (modified method from [66]). Briefly, tracheas were removed and cut longitudinally down the middle of the tracheal cartilage rings. The trachea was placed in the self-made slide. Fluorescent particles (FluoSphere carboxylate, 1.0 µm, Invitrogen, Dreieich, Germany) were applied and particles’ movement was tracking every 0.2 s for 5 min at room temperature using Axiocams 503 mono and ZEN 3.0 (blue edition) software (Zeiss, Oberkochen, Germany). The fluorescent bead velocity was measured by ImageJ software using the Manual tracking plugin.

### 4.7. Patch Clamp 

Cells were patch clamped when grown on coated glass coverslips. Coverslips were mounted in a perfused bath chamber on the stage of an inverted microscope (IM35, Zeiss) and kept at 37 °C. Patch pipettes were filled with a cytosolic-like solution containing (in mM): KCl 30, K-Gluconate 95, NaH_2_PO_4_ 1.2, Na_2_HPO_4_ 4.8, EGTA 1, Ca-Gluconate 0.758, MgCl_2_ 1.03, D-Glucose 5, ATP 3; pH 7.2. The intracellular (pipette) Ca^2+^ activity was 0.1 µM. The bath was perfused continuously with standard bicarbonate-free Ringer’s solution (in mM: NaCl 145, KH_2_PO_4_ 0.4, K_2_HPO_4_ 1.6, Glucose 5, MgCl_2_ 1, Ca-Gluconate 1.3) at a rate of 8 mL/min. Patch pipettes had an input resistance of 2–5 MΩ and whole-cell currents were corrected for serial resistance. Currents were recorded using a patch clamp amplifier EPC9, and PULSE software version 8.65 (HEKA, Lambrecht, Germany) as well as Chart software version 5.5.4 (AD Instruments, Spechbach, Germany). Cells were stimulated with 1 µM ATP in the absence and presence of TRAM34. In regular intervals, membrane voltage (Vc) was clamped in steps of 20 mV from −100 to +100 mV from a holding voltage of −100 mV. The current density was calculated by dividing whole-cell currents by cell capacitance. 

### 4.8. Measurement of [Ca^2+^]_i_^−^

Cells were seeded on coated glass coverslips and loaded with 2 µM Fura-2, AM Ester (Biotium, Hayward, CA, USA) and 0.02% Pluronic F-127 (Invitrogen, Carlsbad, CA, USA) in standard bicarbonate-free Ringer’s solution for 1 h at room temperature. The measurement of intracellular Ca^2+^ concentrations has been described earlier [64]. Cells were then mounted in a thermostatically controlled imaging chamber adapted to an inverted microscope (Axiovert S100, Zeiss, Oberkochen, Germany), maintained at 37 °C and perfused at a rate of 5 mL/min. Fura-2 was excited at 340/380 nm using a high-speed polychromatic illumination system for microscopic fluorescence measurements (Visitron Systems, Puchheim, Germany), and the emission was recorded between 470 and 550 nm using a CoolSnap HQ CCD camera (Roper Scientific, Planegg, Germany/Visitron Systems, Puchheim, Germany). Cells were stimulated with 1, 10, and 100 µM ATP in standard bicarbonate-free Ringer’s solution. Intracellular calcium ([Ca^2+^]_i_) was calculated from the 340/380 nm fluorescence ratio after background subtraction using the formula *[Ca^2+^]_i_ = Kd x (R−R_min_)/(R_max_−R) x (S_f2_/S_b2_),* where *R* is the observed fluorescence ratio. The values *R_max_* and *R_min_* (maximum and minimum ratios) and the constant *S_f2_/S_b2_* (ratio between the fluorescence of free and Ca^2+^-bound Fura-2 at 380 nm) were determined using 2 µM ionomycin (Calbiochem, San Diego, CA, USA), 5 µM nigericin (Sigma-Aldrich, St. Louis, MO, USA), 10 µM monensin (Sigma-Aldrich, St. Louis, MO, USA), and 5 mM EGTA (Carl Roth, Karlsruhe, Germany) to equilibrate intracellular and extracellular Ca^2+^ in intact Fura-2-loaded cells. The dissociation constant (*Kd*) for the Fura-2•Ca^2+^ complex was taken as 224 nM [67]). Control of the experiment, imaging acquisition, and data analysis was conducted with the software package MetaFluor (Universal Imaging, Bedford Hills, New York, NY, USA) [67].

### 4.9. Materials and Statistical Analysis 

All compounds used were of the highest available grade of purity and were bought from Sigma-Aldrich (St. Louis, MO, USA), unless indicated otherwise. Data are shown as individual traces/representative images and/or as summaries with mean values ± SEM, with the respective number of experiments given in each figure legend. The data have been tested for a normal distribution. As the effects have been examined in individual animals, which naturally vary with respect to their responses, outliers have not been removed. Tracings and current–voltage relationships are shown as original data and were not fitted. For the analysis of patch clamp data, PULSE software version 8.65 (HEKA, Lambrecht, Germany) was used. For the quantitative analysis of images and mucociliary clearance, ImageJ software with the manual tracking plugin and Zen (Zeiss, Germany) were used.

For statistical analysis, paired or unpaired Student’s *t*-test or ANOVA were used as appropriate. A *p*-value of < 0.05 was accepted as a statistically significant difference.

## Supplementary Materials

The following supporting information can be downloaded at: https://www.mdpi.com/article/10.3390/ijms23031085/s1.
