# Supplementary material for: Airway Delivery of Hydrogel-Encapsulated Niclosamide for the Treatment of Inflammatory Airway Disease
Source: Int J Mol Sci. 2022 Jan 19;23(3):1085. doi: 10.3390/ijms23031085 (PMC8835663; doi:10.3390/ijms23031085)
Supplement: Supplementary file 1 [file ijms-23-01085-s001.zip › ijms-1510715-supplementary.pdf]

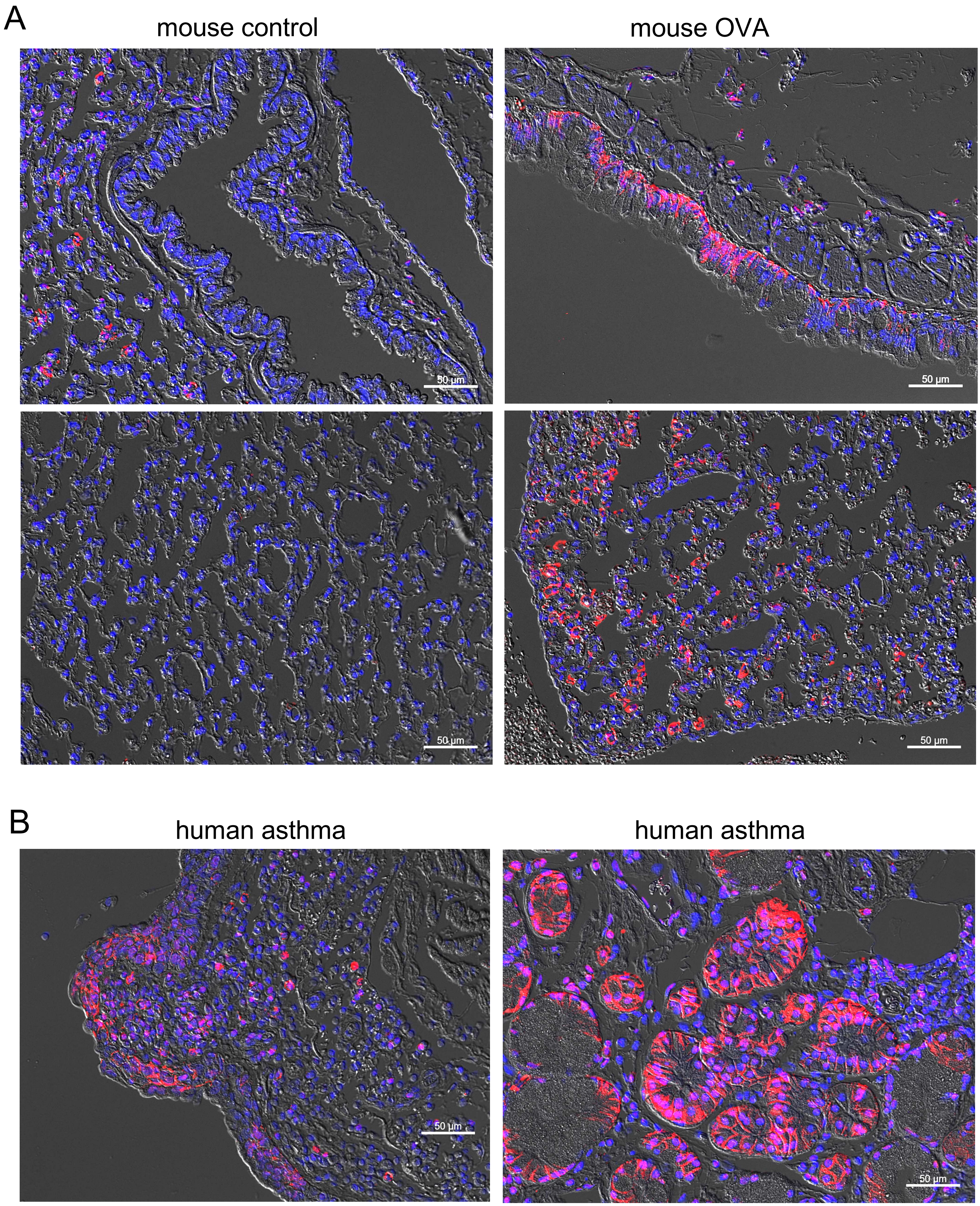

Supplementary Figure 1. *Upregulation of CD44 expression in asthmatic lungs of mouse and human.* A) Upregulation of CD44 in the basolateral membrane of the airway epithelium and in alveolar epithelial cells of ovalbumin-sensitized mice. B) Pronounced expression of CD44 in submucosal glands and lung tissue of asthmatic patient. Bar = 50 µm.
